# Moving forward with forward genetics: A summary of the INFRAFRONTIER Forward Genetics Panel Discussion

**DOI:** 10.12688/f1000research.25369.1

**Published:** 2021-06-08

**Authors:** Asrar Ali Khan, Michael Raess, Martin Hrabe de Angelis

**Affiliations:** 1 INFRAFRONTIER GmbH, Neuherberg / Munich, Bavaria, 85764, Germany

**Keywords:** Forward genetics, mouse models, INFRAFRONTIER, IMPC

## Abstract

In the last few decades, forward genetics approaches have been extensively used to identify gene function. Essentially, forward genetics is the elucidation of the genetic basis of a specific phenotype by screening a population containing random genomic modifications that alter gene function. These approaches have shed light on some essential gene functions in development and disease and have expanded the realm of understanding for genetic disorders. Due to the availability of efficient mutagenesis methods, phenotyping techniques, reliable validation, comprehensive sequence information and translational potential, mouse models are favored for forward genetics approaches. However, in this post-genomic CRISPR-Cas9 era, the relevance and future of forward genetics was brought into question. With more than 7300 mouse strains archived and close interactions with several leading mouse researchers around the world, INFRAFRONTIER - the European Research Infrastructure for mouse models organised a panel discussion on forward genetics at the International Mammalian Genome Conference 2018 to discuss the future of forward genetics as well as challenges faced by researchers using this approach in the current research environment. The commentary presents an overview of this discussion.

## Introduction

**Figure 1.**
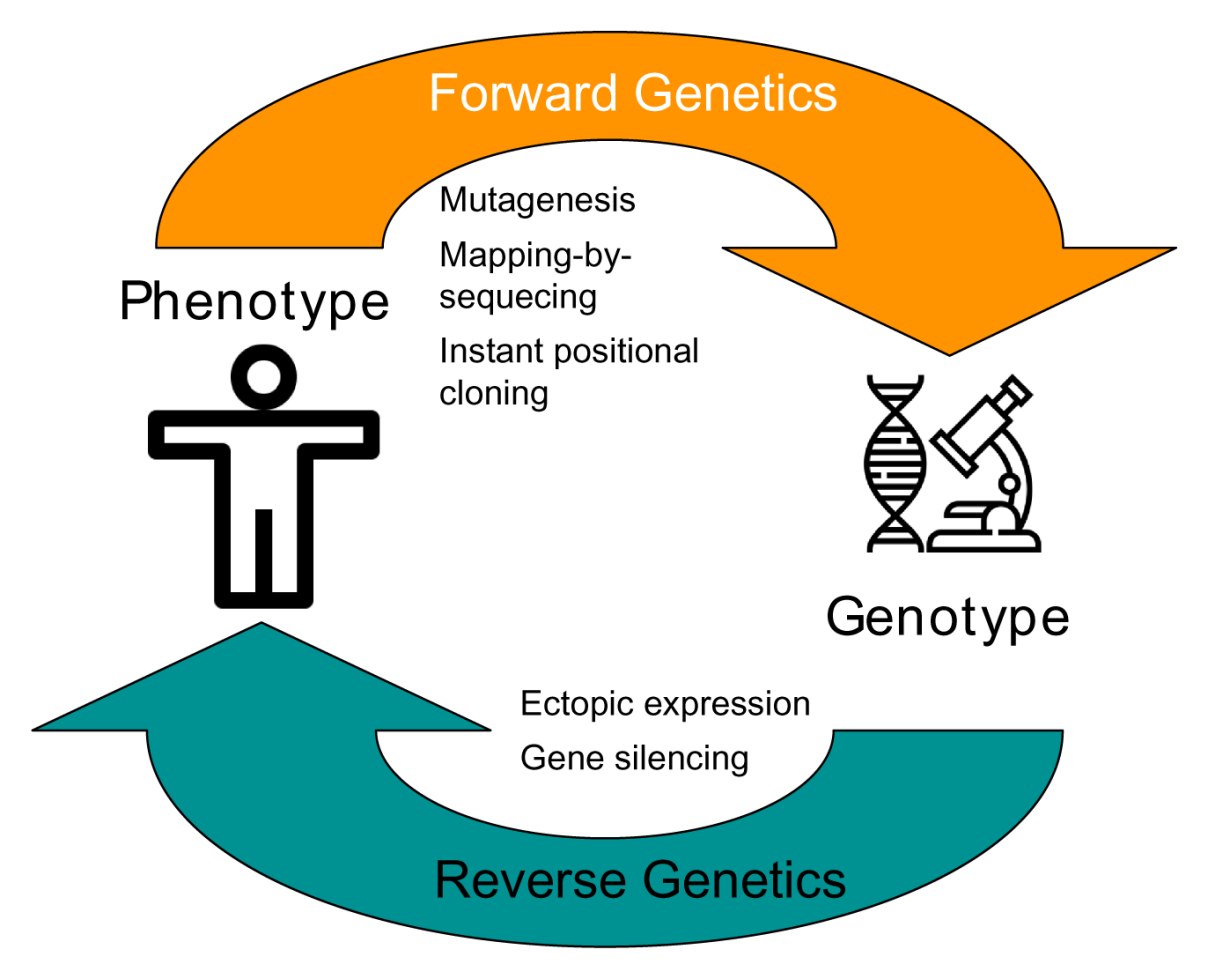
Forward and reverse genetics. Forward genetics (e.g., mutagenesis screens) involves identifying the genetic basis of a phenotype while reverse genetics (e.g., ectopic expression) involves genetic manipulation and study of the resulting phenotype.

Forward genetics is an approach that identifies the genetic basis of a specific phenotype. In medicine, this translates to the discovery of mutations that are responsible for a genetic disease. Contrary to reverse genetics that starts with a specific gene and studies the effects of its altered expression on phenotype, forward genetics uncovers the genes behind a particular phenotype ( Figure 1). Briefly, such an approach starts with a screen to identify a mutant phenotype that is either naturally occurring or artificially induced, for example by N-ethyl-N-nitrosourea (ENU), a potent mutagen ( de Angelis *et al.*, 2000) ( Acevedo-Arozena *et al.*, 2008). Next, the phenotype producing population is mapped followed by mapping of responsible candidate genes and sequencing to find the causative mutation. In the final step, the candidate mutation is validated using genetic engineering approaches.

**Figure 2.**
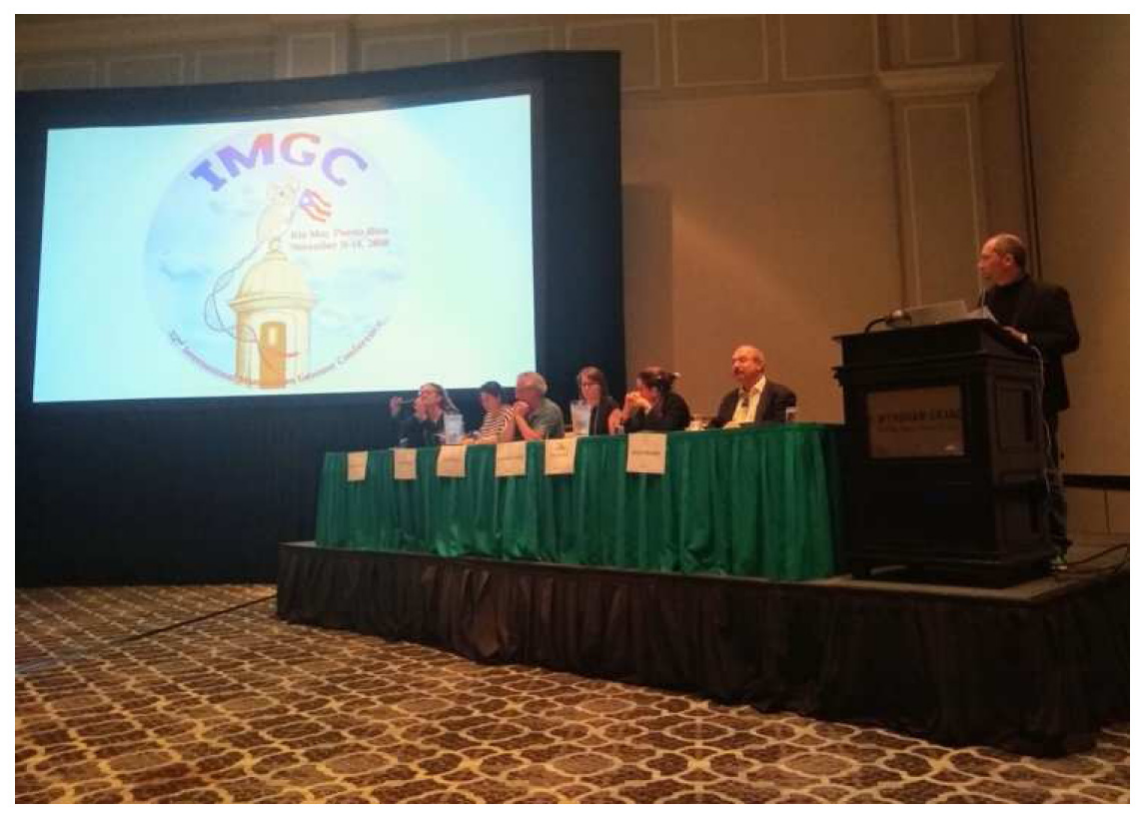
The INFRAFRONTIER Forward Genetics Panel Discussion at the IMGC 2018.

Over the years, forward genetics has made significant contributions to human genetics research. For example, the etiology of several monogenic diseases like Rett syndrome ( Buchovecky *et al*., 2013) and Huntington’s disease ( Macdonald *et al*., 1993) have been successfully discovered using forward genetics approaches. The mutations responsible for complex phenotypes and disorders like obesity ( Zhang *et al*., 1994), infertility ( Hu *et al*., 2019) and circadian rhythm dysregulation ( Vitaterna *et al*., 1994) were also successfully identified using model organisms like mice. The biggest advantage of this approach is its unbiased nature for establishing a clear relationship between a mutation and disease phenotype.

At a scientific review meeting of the Helmholtz Center Munich in February 2018, an interesting discussion was initiated about the current status and future directions of forward genetics. ‘Where does forward genetics fit in other technologies in this post-genomic era and what is its relevance?’. This initial discussion led to the organization of the Forward Genetics Panel Discussion at the International Mammalian Genome Conference 2018 (IMGC 2018). The objective of the panel discussion was to explore the current state and future of forward genetics in the light of the recent developments in (functional) genomics and data-driven science. The panellists consisted of international leaders in the field of mouse genetics and pioneers in forward genetics approaches. The panel discussion was chaired by Prof. Dr. Martin Hrabě de Angelis.

The panel discussion was preceded by a lecture from Prof. Dr. Bruce Beutler (Nobel Prize in Physiology or Medicine, 2011) on his ongoing state-of-the-art forward genetics screens to uncover mutations leading to immunological phenotypes. This entire session on Forward Genetics at the IMGC 2018 was hosted by INFRAFRONTIER via the IPAD-MD project.

## Panellists

**Prof. Dr. Bruce Beutler**, director of the Center for the Genetics of Host Defense (UT Southwestern Medical Center), has been one of the foremost leaders in the field of forward genetics and has made several key discoveries in immunology using this approach. Currently, his team conduct robust and automated forward genetics screens to identify genes responsible for specific physiological processes like immunity, metabolism, developmental and neurobehavioral functions.

**Prof. Dr. Monica Justice** is the head of the Genetics and Genome Biology Program at the Hospital for Sick Children (SickKids). Prof. Justice pioneered the use of ENU-based chemical mutagenesis approaches in mice and she has recently shown DNA damage response contributes to the pathology of Rett Syndrome.

**Dr. Laura Reinholdt** is an associate professor at the The Jackson Laboratory and has led the cloning of several ENU alleles responsible for aberrant meiotic chromosome dynamics under the Reprogenomics ENU program. Her group is widely interested in the development and application of both forward and reverse genetic approaches for understanding the etiology of genome variation and its role in health and disease.

**Prof. Dr. David Beier**, director of the Center for Developmental Biology and Regenerative Medicine at the Seattle Children's Research Institute, has been on the forefront of several major developments in the genetic analysis of model organisms including the application of ENU mutagenesis for developmental investigation and for sequence-based analysis.

**Prof. Dr. Nadia Rosenthal** is the scientific director of The Jackson Laboratory and a renowned expert in the use of mice for targeted mutagenesis in the study of muscle development, disease and repair. An integral part of EUCOMM, the European Conditional Mouse Mutagenesis Program, she coordinated the selection and production of new Cre driver strains for the international mouse genetics community.

**Dr. Ruth Arkell** heads the Early Mammalian Development Laboratory at the John Curtin School of Medical Research (ANU) and has conducted several forward genetics genome-wide ENU mutagenesis screens at MRC Harvell and ANU. She now focuses on the genetic mechanisms that control mammalian gastrulation and the consequences of incorrect gastrulation.

**Prof. Dr. Martin Hrabě de Angelis**, director of the European Mouse Mutant Archive (EMMA) and the Institute of Experimental Genetics at the Helmholtz Center Munich, is a strong proponent of forward genetics and has made several prominent discoveries in the field of genetics using ENU mutagenesis forward genetics screens.

## Contribution of forward genetics towards understanding the genetic basis of human disease

According to the panellists, there was no one particular forward genetics contribution that was the important towards the better understanding of human diseases and mammalian physiology. Some of the notable contributions of forward genetics were the discovery of Toll-like receptor (TLR)-4 as the lipopolysaccharide sensor ( Poltorak *et al*., 1998) and discovery of *Clock* gene as the central regulator of mammalian circadian rhythm ( Daxinger *et al*., 2013), discovery of early mammalian development genes ( Anderson, 2000) and models for metabolic bone diseases ( Sabrautzki *et al*., 2012).

In their view, forward genetics is the re-evaluated application of classical mendelian genetics because it also deals with the heritability of quantifiable traits. It has gained more importance in the last few decades as the actual complexity of the genome has started to become evident.

## Relevance of forward genetics in the post-genomic era

Forward genetics is remains highly relevant in the post-genomic era. Human geneticists are realising the importance of mouse models replicating the exact mutation found in human patients whereas previously they heavily relied on sequencing, association studies and reverse genetics to understand complex human diseases. The impact of environment and diet on diseases is widely acknowledged now. In addition, in most cases it is not a single gene but a network of genes that is responsible for a disease. Therefore, more sophisticated human diseases models are needed to accurately emulate complex human pathologies. One of the main advantages of forward genetics approaches is their unbiased nature, requiring no prior knowledge of a biological pathway. In addition, a wide spectrum of mutations is possible using mutagens that can create a variety of alleles (hypomorphicm hypermorphic, neomorphic and conditional) alleviating the problems of lethality. With their unbiased nature, forward genetic approaches can determine which genetic regulatory networks have pathogenic consequences and also employ complex mouse models which would be immensely helpful to human geneticists.

## Advancements in forward genetics

In the last few decades, several advancements have led to significant progression of forward genetic screens. Some of them are listed below:

**Positional cloning:** The process of positional cloning, i.e. the identification of the causative mutation, was expedited by the publication of the annotated mouse genome in 2002 and sequencing of whole mammalian exomes. Previously, such sequencing endeavours required more than 9 years to complete and now can be accomplished in a few weeks.

**Genetic mapping:** Another breakthrough was the ‘instant positional cloning’ technique that could resolve disease phenotypes almost instantaneously ( Wang *et al*., 2015) thereby removing the bottleneck of genetic mapping. High-throughput sequencing (HTS) technologies have expedited forward genetic screens with rapid mutation discovery (as reviewed by Simon *et al*., 2012). An approach called mapping-by-sequencing uses next-generation sequencing to simultaneously map and identify causal mutation by sequencing bulk populations of recombinant offspring ( Schneeberger *et al*., 2009). A number of recent advancements of this approach have made forward genetic screens more accessible. These advancements include methods that are independent of reference genome sequences, genetic crosses and any kind of linkage information (as reviewed by Schneeberger, 2014). The use of whole-genome sequencing (WGS) has also made it possible to study mutant lines in an inbred genetic background and identify elusive genetic modifiers ( Geister *et al*., 2018).

**Mutant production:** CRISPR-Cas9 genome editing has been used in forward genetics screens to create genome-wide mutant libraries because to its easier scalability. In addition, unlike chemical mutagens or radiation, CRISPR allows the generation of mutant libraries with known mutation sites. On one side, the development and use of inbred mice also made forward genetic screens easier. These strains possess a near overall homozygosity in their genetic loci making the identification of the causative mutation faster and cheaper. While on the other side, genetic screens have also started to utilize genetically diverse mouse strains that are generated by out-breeding and not by mutagenesis. These offer the advantage of replicating the genetic diversity found in humans to a certain extent and also capture structural genetic variants that are missed by mutagenesis ( Saul *et al*., 2019).

**Phenotype screening:** Several new technologies have greatly improved the efficacy of phenotype screening methods used in forward genetics screens. These include induced pluripotent stem cells, 3D-culture systems and organ-on-a-chip. In addition, the use of *in vivo* models, like fruit flies, *C. elegans* and mice, in phenotypic screens have opened up new avenues for forward genetics by standardising phenotyping pipelines. These technologies and advancements have enabled forward genetic screens to realistically recapitulate human disease biology.

**Validation:** The use of CRISPR also enabled efficient validation of disease mutations by the rapid generation of specific mouse models reproducing the disease phenotype. 

Thus, the last few decades of advancements in genome editing, *in vitro* and *in vivo* models, and sequencing technologies collectively propelled forward genetic screens.

## Future of forward genetics

Human geneticists primarily rely on mapping of genetic variants (genome-wide association studies) to determine pathogenic genetic changes. As mentioned previously, forward genetics is invaluable in this regard and can provide an efficient way to molecularly assess and validate such mutations thereby bridging the relationship between functional genetic variation and human diseases.

CRISPR-Cas9 has been a disruptive technology in field of genetic engineering. The efficiency, accuracy and ease of operation compared to previous technologies has also brought CRISPR-Cas9 into functional genomics screens as well. Not only has it improved reverse genetics, i.e. the validation of discovered mutations (mentioned above), but several studies have also shown its usefulness in forward genetics screens. As reviewed by Sharma & Petsalaki (2018), these pooled CRISPR-Cas9 screens offer the possibility to investigate a very large number of genetic changes in one screen.

Forward genetics screens are in fact promising classical genetics tools that are customizable and easy to use. This aspect is especially enticing for various research groups to undertake smaller customized screens that are specifically tailored to a scientific question. Subsequent services like cloning, phenotyping, genetic mapping and sequencing can be supported by larger core centres or infrastructures.

## Challenges (technical and financial) in the field of forward genetics

As stated earlier, positional cloning was a major rate limiting step in forward genetics screen which has been overcome with the ‘instant positional cloning’ method. The speed and affordability of current-generation sequencing technologies has also greatly helped in this direction. However, these advancements have led to the generation of large amount of genotypic and phenotypic data increasing the demand for the mechanistic analyses needed to make sense of precisely how specific mutations lead to specific phenotypes. As mentioned earlier, CRISPR is a promising tool for forward genetics. However, its use in forward genetics can still be improved by minimising off-target effects, and by targeting isoforms or splice variants and non-coding sequences like regulatory elements.

One of the main challenges faced in forward genetics today is the competition for funding from human geneticists with funding agencies arguing that animal models like mice are not suitable tools to discover, validate and study disease causing mutations. As mentioned earlier, human geneticists require allele-specific models for translational research of human diseases that can also be used for preclinical studies. The generation of such ‘synthetic complex disease models’ is currently only possible in mice.

One aspect that still requires improvements is the automated handling of animal models in these screens as they involve breeding and housing of large number of mutant animals and their subsequent progeny which is labour-, time-, and cost-intensive. 

## Possible cooperative efforts from the forward genetics community

It was agreed upon that a collective effort is required to bring back forward genetics into the limelight and promote its use in present and future biomedical research. One such cooperative effort would be to develop a precision model generation and robust phenotyping pipeline for characterizing human functional genetic variation in mouse models. This pipeline could be used by human geneticists to investigate pathogenic allelic variations and enable mouse geneticists to provide valuable and reliable support to human geneticists.

More focus should be placed on gene regulatory pathways and not on individual genes, especially when involving model organisms. This would circumvent the problem of missing orthologs in humans. In addition, more effective ways need to be applied to translate molecular mechanisms to phenotypes.

Apart from deciphering the relationship between functional genetic variation and disease, it is also important to focus on the biology of the disease as a whole.

## Closing remarks

It was evident from the panel discussion that forward genetics is still valuable to human genetics and is needed to understand the genetic basis of human diseases. In addition, animal models (especially mouse models) hold enormous potential when combined with forward genetics screens and can efficiently complement human genetics research in the form of precise disease models. Recent advances in genome editing, sequencing technologies and mutant generation have made forward genetics screens accessible to the wider community of biomedical researchers. Consequently, this needs to be effectively communicated to the scientific community and policy officials. The panellists agreed that a review or commentary highlighting the outcomes of the panel discussion would be a logical next step. Another tangible outcome was the renewed focus on bringing together and strengthening interactions between forward, mouse and human geneticists in upcoming INFRAFRONTIER stakeholder meetings and conferences.

## Data availability

No data are associated with this article.

## References

[ref-1] Acevedo-Arozena A Wells S Potter P : ENU mutagenesis, a way forward to understand gene function. *Annu Rev Genomics Hum Genet.* 2008;9:49–69. 10.1146/annurev.genom.9.081307.164224 18949851

[ref-2] Anderson KV : Finding the genes that direct mammalian development : ENU mutagenesis in the mouse. *Trends Genet.* 2000;16(3):99–102. 10.1016/s0168-9525(99)01921-6 10689347

[ref-3] Buchovecky CM Turley SD Brown HM : A suppressor screen in *Mecp2* mutant mice implicates cholesterol metabolism in Rett syndrome. *Nat Genet.* 2013;45(9):1013–1020. 10.1038/ng.2714 23892605 PMC3837522

[ref-4] Daxinger L Harten SK Oey H : An ENU mutagenesis screen identifies novel and known genes involved in epigenetic processes in the mouse. *Genome Biol.* 2013;14(9):R96. 10.1186/gb-2013-14-9-r96 24025402 PMC4053835

[ref-5] de Angelis MHH Flaswinkel H Fuchs H : Genome-wide, large-scale production of mutant mice by ENU mutagenesis. *Nat Genet.* 2000;25(4):444–447. 10.1038/78146 10932192

[ref-6] Geister KA Timms AE Beier DR : Optimizing Genomic Methods for Mapping and Identification of Candidate Variants in ENU Mutagenesis Screens Using Inbred Mice. *G3 (Bethesda).* 2018;8(2):401–409. 10.1534/g3.117.300292 29208648 PMC5919724

[ref-7] Hu J Lessard C Longstaff C : ENU-induced mutant allele of *Dnah1*, *ferf1*, causes abnormal sperm behavior and fertilization failure in mice. *Mol Reprod Dev.* 2019;86(4):416–425. 10.1002/mrd.23120 30734403

[ref-8] Macdonald ME Ambrose CM Duyao MP : A novel gene containing a trinucleotide repeat that is expanded and unstable on Huntington's disease chromosomes. The Huntington's Disease Collaborative Research Group. *Cell.* 1993;72(6):971–983. 10.1016/0092-8674(93)90585-E 8458085

[ref-9] Poltorak A He X Smirnova I : Defective LPS signaling in C3H/HeJ and C57BL/10ScCr mice: mutations in *Tlr4* gene. *Science.* 1998;282(5396):2085–2088. 10.1126/science.282.5396.2085 9851930

[ref-10] Sabrautzki S Rubio-Aliaga I Hans W : New mouse models for metabolic bone diseases generated by genome-wide ENU mutagenesis. *Mamm Genome.* 2012;23(7–8):416–430. 10.1007/s00335-012-9397-z 22527485 PMC3401305

[ref-11] Saul MC Philip VM Reinholdt LG : High-Diversity Mouse Populations for Complex Traits. *Trends Genet.* 2019;35(7):501–514. 10.1016/j.tig.2019.04.003 31133439 PMC6571031

[ref-12] Schneeberger K : Using next-generation sequencing to isolate mutant genes from forward genetic screens. *Nat Rev Genet.* 2014;15(10):662–676. 10.1038/nrg3745 25139187

[ref-13] Schneeberger K Ossowski S Lanz C : SHOREmap: simultaneous mapping and mutation identification by deep sequencing. *Nat Methods.* 2009;6(8):550–551. 10.1038/nmeth0809-550 19644454

[ref-14] Sharma S Petsalaki E : Application of CRISPR-Cas9 Based Genome-Wide Screening Approaches to Study Cellular Signalling Mechanisms. *Int J Mol Sci.* 2018;19(4):933. 10.3390/ijms19040933 29561791 PMC5979383

[ref-15] Simon MM Mallon AM Howell GR : High throughput sequencing approaches to mutation discovery in the mouse. *Mamm Genome.* 2012;23(9–10):499–513. 10.1007/s00335-012-9424-0 22991087 PMC3724459

[ref-16] Vitaterna MH King DP Chang AM : Mutagenesis and mapping of a mouse gene, Clock, essential for circadian behavior. *Science.* 1994;264(5159):719–725. 10.1126/science.8171325 8171325 PMC3839659

[ref-17] Wang T Zhan X Bu CH : Real-time resolution of point mutations that cause phenovariance in mice. *Proc Natl Acad Sci U S A.* 2015;112(5):E440–449. 10.1073/pnas.1423216112 25605905 PMC4321302

[ref-18] Zhang Y Proenca R Maffei M : Positional cloning of the mouse *obese* gene and its human homologue. *Nature.* 1994;372(6505):425–432. 10.1038/372425a0 7984236

