# Supplementary material for: Moving forward with forward genetics: A summary of the INFRAFRONTIER Forward Genetics Panel Discussion
Source: F1000Res. 2021 Jun 8;10:456. [Version 1] doi: 10.12688/f1000research.25369.1 (PMC8634052; doi:10.12688/f1000research.25369.1)
Supplement: Peer review history

# Peer review: Reviewer response for version 1

**DOI:** 10.5256/f1000research.27993.r97637

**Published:** 2021-11-30

**Authors:** Camron D Bryant

**Affiliations:** 1 Laboratory of Addiction Genetics, Department of Pharmacology and Experimental Therapeutics and Psychiatry, Boston University School of Medicine, Boston, MA, USA

This is a much needed discussion/summary as it addresses the ongoing backlash against forward genetics of complex traits and disease models that human geneticists have instigated against quantitative geneticists that employ model organisms.

 Many of my comments below are with regard to proofing, with a few conceptual comments/suggestions

1. Definition of “forward genetics” at the beginning seems ENU-centric and does not sound like it includes QTL and GWAS. “random genomic modifications that alter gene function” (includes both naturally occurring and artificially induced).
2. There must be a word missing here: “the phenotype producing population is mapped…” the phenotype producing population *variance* is mapped? Also, too many “mapped”s in this sentence.
3. “The mutations responsible for complex phenotypes and disorders…” I would change to “Mutations contributing to complex phenotypes and disorders…” (We do not know the full genetic architecture yet of any complex disease or phenotype.)
4. “there was no particular forward genetics contribution that was the important” – should say “the most important”?
5. “quantifiable traits” should be “quantitative traits”
6. “in their view, forward genetics is the…” should be “in their view, contemporary forward genetics comprises….”
7. “Forward genetics is remains…” should be “Forward genetics remains…”
8. “Therefore, more sophisticated human diseases models…” should be “…more sophisticated models of human diseases….”
9. “In addition, a wide spectrum of mutations is possible using mutagens that can create a variety of alleles…” This sentence omits natural alleles that also comprise a wide variety of non-lethal yet sometimes large-effect mutations on phenotypes
10. Another advantage of forward genetics in model organisms is the detection of epistasis and the study of its biological interactions and mechanisms. Modifiers were mentioned once under the section “Mutant Production”
11. While, mapping with highly diverse, outbred populations was mentioned, mapping with less diverse, yet more highly recombinant populations such as commercial outbred was omitted. These have the advantage of producing even higher resolution QTLs. Also, the use of reduced complexity crosses (RCCs) between near isogenic strains was omitted. There are several advantages to RCCs (speed, two clear genetic backgrounds to demonstrate necessity and sufficiency, etc.)
12. There is definitely a bias in this article toward the mutagenesis side of forward genetics (e.g., the repeated discussion/mention of “instant positional cloning” but positional cloning is not instant for QTL/GWAS studies of natural genetic and phenotypic variation. High-resolution QTLs is an advancement. Positional cloning has become more precise with WGS as one can precisely select a panel or markers to monitor recombination events for fine mapping/cloning
13. In my opinion, no specific concrete examples were elaborated upon in any level of depth to make the case that forward genetics in model organisms is relevant and offers advantages over discovery based genetics in humans. I applaud the effort behind arranging this panel and summarizing the discussion but I think there is room for making a stronger case for forward genetics in model organisms than currently stands. Perhaps this brief article is not the venue for achieving this goal as it seems to represent what was actually discussed at the panel rather than what could have been discussed or what should have been discussed in greater detail.

Is the topic of the opinion article discussed accurately in the context of the current literature?

Yes

Are arguments sufficiently supported by evidence from the published literature?

Partly

Are all factual statements correct and adequately supported by citations?

Yes

Are the conclusions drawn balanced and justified on the basis of the presented arguments?

Yes

Reviewer Expertise:

addiction genetics in rodent model organisms

I confirm that I have read this submission and believe that I have an appropriate level of expertise to confirm that it is of an acceptable scientific standard, however I have significant reservations, as outlined above.

# Peer review: Reviewer response for version 1

**DOI:** 10.5256/f1000research.27993.r97639

**Published:** 2021-11-03

**Authors:** Anton Wutz, Corinne Kaufmann

**Affiliations:** 1 Swiss Federal Institute of Technology , Zürich, 8093, Switzerland

The manuscript entitled "Moving forward with forward genetics: A summary of the INFRAFRONTIER Forward Genetics Panel Discussion" provides a summary and visions arising from the panel discussion of the INFRAFRONTIER program on forward genetics. The authors were panelists at the occasion and have submitted a well-structured synopsis that can be used as a reference for the present state and future directions. As such it will be useful for transparently documenting for the interested scientific community and policy makers in Europe. Although the article is well written, some details below could be considered by the authors for further improving the readability.

**Specific points:**

1. The title raises expectations of insights into how to perform forward genetics but the article falls a bit short of delivering on this. The authors could consider including more information on new technologies that might be incorporated into forward genetics such as CRISPR Cas systems that have seen tremendous success in the past few years. Is it conceivable that both field synergies such that onboarding of base editors into forward genetics could provide an opportunity for developing the CRISPR applications further?
2. The present version of the article does not make it easily accessible to a wide readership as to the commendable accomplishments in the past. For improving the impact of the manuscript it could be suggested to provide a concrete example. Maybe from one of the works of the panelists.
3. Research on the organism level has the great advantage of allowing to study physiology questions. This appears to come at of cost and one wonders if over time methods and technologies have come forth that would make the endeavour more efficient going into the future. Could the authors briefly introduce the limitations and bottlenecks of forward genetics in mice at present? This could then serve to position the present proposal of future-forward genetic studies. Citing current research of new ways for introducing mutations more efficiently or unmasking phenotypes would provide the reader with concrete ideas and make the text more exciting.
4. In general, it seems clear that genetics is an important method for gaining insights into disease and developmental biology. The text appears to focus specifically on forward genetics. On one hand, this appears to artificially limit discussion and on the other hand, the authors themselves cite applications that seem to be not clearly forward genetically in nature. Page 4, right column, beginning of the last paragraph discusses the analysis of human disease mutations in mice. This likely could be seen as a reverse genetics approach. It would appear important to specifically define this in order to avoid confusing readers.
5. Page 5, right column, beginning of the last paragraph briefly refers to CRISPR technology and raises current problems. It would be interesting to briefly state how this system could be used and a promising tool as it appears that limitations might be overcome quickly in this rapidly progressing field. A comparison of the advantages and disadvantages of both systems could be informative for the reader.
6. In the introduction, the authors refer to the MeCP2 mutation in mice as a model of human RETT syndrome. It might be wise to consider more careful wording as in strict genetics terms the disease is caused by heterozygosity in female patients whereas it is a homozygous / hemigynous mutation that leads to motor impairment in mice. Maybe a sentence reconciling the phenotypic and genetic differences could be included for factual correctness.
7.  As the manuscript is considered as a policy document it would appear of importance to position the proposed method against related approaches. Given the enormous efforts in time and cost for maintaining mice, the authors might wish to explain the big advantages over culture-based approaches. Firstly, redundancy among genes can be unmasked by observing the phenotype in different tissues where redundant factors might not be masked. Secondly, complex physiological interactions can only be studied in the tissues and organs of the organism and therefore the proposed approach would extend competing approaches that aim to identify the molecular basis of human disease in culture systems. Would a proposal based on large numbers of animals not appear untimely without considering alternative approaches?

Is the topic of the opinion article discussed accurately in the context of the current literature?

Yes

Are arguments sufficiently supported by evidence from the published literature?

Yes

Are all factual statements correct and adequately supported by citations?

Partly

Are the conclusions drawn balanced and justified on the basis of the presented arguments?

Partly

Reviewer Expertise:

Mouse genetics, dosage compensation, development, stem cells

We confirm that we have read this submission and believe that we have an appropriate level of expertise to confirm that it is of an acceptable scientific standard, however we have significant reservations, as outlined above.

# Peer review: Reviewer response for version 1

**DOI:** 10.5256/f1000research.27993.r87083

**Published:** 2021-06-30

**Authors:** Martin S Denzel

**Affiliations:** 1 Max Planck Institute for Biology of Ageing, Cologne, Germany

The commentary by Khan *et al*. is an interesting and timely reminder about the strengths of forward genetic screens in mice and nicely points out recent developments in the field that assure that such screens remain valuable in understanding gene function and disease mechanisms.

 I have a few suggestions below:

1. Abstract: “in development and diseases”, I would suggest adding “in the maintenance of homeostasis”.
2. The section: “Briefly, such an approach starts with a screen to identify a mutant phenotype that is either naturally occurring or artificially induced, for example by N-ethyl-N-nitrosourea (ENU), a potent mutagen” should also mention transposons and CRISPR as means to introduce mutations in forward screens.
3. Figure 2 is not very useful – I would suggest making a figure with portrait pictures of the panelists that could nicely go with their introductions.
4. Contributions of… section: The first sentence “According to the panelists, there was no one particular forward genetics contribution that was the important towards the better understanding of human diseases and mammalian physiology” is not clear and perhaps it might be a better read to start the section with a positive notion.
5. The following sentence should be re-phrased for clarity: “CRISPR-Cas9 genome editing has been used in forward genetics screens to create genome-wide mutant libraries because to its easier scalability.”
6. One way to cope with the challenges of forward genetic screens in mice it to use the same methodologies (ENU, crispr etc) in cultured cells, and the use of haploid cells for mutagenesis (as described by the Jackson lab, PMID: 27820796 ^1^ and my lab PMID: 29515774 ^2^ and PMID: 32690882 ^3^) provides a serious alternative for cellular phenotypes.  

Typos:

- In the intro section for Prof B Beutler, “Currently, his team conduct robust...” should be “Currently, his team conducts robust…”
- “Therefore, more sophisticated human diseaseS models are needed to accurately emulate complex human pathologies.” should be “Therefore, more sophisticated human disease models are needed to accurately emulate complex human pathologies.”
- “As stated earlier, positional cloning was a major rate limiting step in forward genetics screen which has been overcome with the ‘instant positional cloning’ method.” should be  “As stated earlier, positional cloning was a major rate limiting step in forward genetic screens, which was overcome by ‘instant positional cloning’.”
- “…hold enormous potential when combined with forward genetics screens” should be “…hold enormous potential when combined with forward genetic screens”

Is the topic of the opinion article discussed accurately in the context of the current literature?

Yes

Are arguments sufficiently supported by evidence from the published literature?

Yes

Are all factual statements correct and adequately supported by citations?

No

Are the conclusions drawn balanced and justified on the basis of the presented arguments?

Yes

Reviewer Expertise:

forward genetic screens, aging

I confirm that I have read this submission and believe that I have an appropriate level of expertise to confirm that it is of an acceptable scientific standard.

## References

[1] : Genome-wide genetic screening with chemically mutagenized haploid embryonic stem cells. *Nat Chem Biol* .2017;13(1) : 10.1038/nchembio.2226 12-14 10.1038/nchembio.2226 27820796PMC5164930

[2] : Unbiased compound-protein interface mapping and prediction of chemoresistance loci through forward genetics in haploid stem cells. *Oncotarget* .2018;9(11) : 10.18632/oncotarget.24305 9838-9851 10.18632/oncotarget.24305 29515774PMC5839405

[3] : Bortezomib resistance mutations in PSMB5 determine response to second-generation proteasome inhibitors in multiple myeloma. *Leukemia* .35(3) : 10.1038/s41375-020-0989-4 887-892 10.1038/s41375-020-0989-4 32690882PMC7932915
